# Territorial expansion and parasitic zoonoses: the pampas gray fox in Argentina as a case study and its educational potential for prevention

**DOI:** 10.3389/fvets.2026.1896506

**Published:** 2026-08-20

**Authors:** Damian Alberto Lampert, Matias Russo

**Affiliations:** 1 Universidad Nacional de Quilmes, Departamento de Ciencia y Tecnología, Grupo de Investigación en Enseñanza de las Ciencias (GIECIEN), Quilmes/Buenos Aires, Argentina; 2 Facultad de Ciencias Veterinarias, Universidad Nacional de La Plata, La Plata/Buenos Aires, Argentina

**Keywords:** critical thinking, Pampas gray fox, STS education, urbanization, zoonoses

Many authors have asserted that contemporary urban areas are undergoing rapid spatial and demographic growth, but this growth stems from deficient planning, generating numerous environmental conflicts. Among these conflicts is the fragmentation of scarce green spaces, leading to the isolation of certain native animals, a limited capacity to assimilate pollution, and ecosystem fragility that is difficult to repair (1). Consequently, species exhibit discontinuous distribution patterns resulting from spatial variations in environmental conditions, leading to continuous changes in territorial structure and the formation of a heterogeneous landscape (2).

According to Santos and Tellería (2), these processes have numerous consequences: regional habitat loss and the resulting reduction in the size of affected organism populations, which decreases the regional density of species; an increase in the distance between fragments, hindering the exchange of individuals between isolated populations and limiting the replenishment of these populations; and greater exposure of the fragmented habitat to multiple interferences from surrounding habitats (habitat matrix), resulting in a growing “edge effect” that causes a decline in habitat quality, thereby affecting the survival of populations residing in the fragments. Habitat destruction, fragmentation, and degradation break up animal and plant populations into small, isolated groups. If this problem continues unchecked, the end result is an impoverished landscape where many of the original species disappear (2).

Considering the “One Health” approach, it is important to understand that environmental impacts and their consequences for animal habitats also create problems for the surrounding communities or even for those living alongside them. This is because human–animal relationships become more complex with each generation. Although animals continue to fulfill a utilitarian role in human life (e.g., the food industry, animal breeding, and animal sales), they are affected by the environmental controls implemented by humans due to their susceptibility (3). This leads to the normalization, evolution, and restriction of human–animal interaction with species that should not be in such circumstances. These species are influenced by territorial transformations, human convenience, and also the affectionate relationships that develop with them because of the insensitivity caused by convenience, ignorance, or ideology (2, 3). However, this also has negative consequences for human beings. As Lampert et al. (4) state, these situations carry risks for the community, such as zoonoses, which are consequences that arise from compromising or threatening the environment. This creates conditions that foster a breeding ground for disease by making environments unhealthy for life, since human health cannot be considered independently of the health of other organisms. This highlights the importance of a comprehensive approach that considers all the components of One Health (4).

Understanding the importance of the integrity of all actors within a community provides a different perspective on the relevance of territorial transformations and urban planning to public health risks. The presence of canids in urban areas is directly related to anthropogenic processes. Temporal interactions and competition among species also arise depending on variables such as housing density and forest cover (5). The consequences of urbanization, which accompany changes in land use, the incorporation of substances to enhance soil productivity, and habitat loss and fragmentation, alter the distribution of carnivorous mammals (6). Furthermore, the potential consumption of these animals by humans is another factor that may influence the presence of wild mammals in areas currently inhabited by humans (6, 14). All these factors contribute to the loss of biodiversity, which is accompanied by the loss of natural habitats, the reorganization of communities (6), and nutritional and metabolic disorders associated with the consumption of ultra-processed foods by humans (14).

Human activities that influence the interaction with canids are affected by the animals' sensitivity to human presence. This sensitivity to humans was demonstrated during the COVID-19 pandemic. One study found that gray foxes (*Urocyon cinereoargenteus)*, red foxes (*Vulpes vulpes*), and coyotes (*Canis latrans*) modified their daily activities in both urban and rural areas (7). Gray foxes changed their daily activities in rural areas, while coyotes altered theirs in urban areas (7). Gray foxes were the canids that exhibited the greatest change in daily activity during the pandemic (7).

For example, the Pampas gray fox (*Lycalopex gymnocercus*) is one of the most widespread and abundant wild canids in Argentina. However, it is common to see news reports of this species being found in urban areas. This may pose a potential risk for the transmission of parasitic zoonotic diseases. A study conducted in the Province of Buenos Aires found that, among 142 foxes found dead and/or captured, the digestive tracts harbored 16 species of helminths (4 cestodes, 1 trematode, and 11 nematodes) and 9 species of protozoa (8). Another study reported the presence of Neospora caninum in the animal. This is of great concern because it is a protozoan that causes abortions and reproductive failures in livestock, resulting in significant economic losses (9). In Argentina, there are numerous reports of Pampas gray foxes occurring in urban and rural areas inhabited by people. Urban expansion into natural habitats is leading to increasingly close contact between humans and native wildlife, which can facilitate the transmission of zoonotic diseases. Given this situation, education plays a crucial role in disease prevention.

The Pampas gray fox is of great ecological importance. Its role as a predator, scavenger, frugivore, and seed disperser has contributed significantly to the development of diverse biomes (10). This animal is a clear example of the importance of One Health education, based on the One Health approach. From an educational perspective, the Science, Technology, and Society (STS) approach is a valuable tool for fostering critical thinking in students, contextualizing learning, and enabling the community to understand different perspectives on this animal. Lampert (14) wrote a book titled “When the Jungle Sits Down to Eat,” which analyzes how the welfare of coatis (*Nasua nasua*), the prevalence of zoonoses, and their behavior are affected by human interaction in Iguazu National Park (Misiones Province, Argentina). Previous research has demonstrated that classroom-based approaches to animal ecology, behavior, welfare, and health risks foster the development of critical thinking skills to prevent harmful human–animal interactions (14). Although this research focused on coatis, it is essential to address this educational topic for each species that enters urban areas, emphasizing prevention. Furthermore, various interviews and studies have shown that people become more aware of the potential negative effects of these interactions on animals, rather than focusing solely on themselves. Therefore, One Health education for prevention should focus not only on people but also on the risks faced by these species.

For the Pampas gray fox, the issue of domestic dogs (*Canis familiaris*) is a factor within the broader context of habitat alteration. It is important to note that foxes are evolutionarily similar to domestic dogs, not only in terms of anatomy and physiology but also in terms of behavior (11). Peri-urban areas can serve as potential hotspots for pathogen exposure among dog and fox populations; consequently, both domestic dogs and wild foxes may be exposed to the same diseases. The presence of dogs in livestock and rural areas, combined with the encroachment of foxes into these zones (driven by habitat fragmentation and poor waste management), turns these interactions into a transmission pathway for pathogens of veterinary and public health significance (11).

Recent research describes a case in Brazil where domestic dogs may act as bridge hosts for *Rickettsia parkeri*, transporting infected tick vectors from areas shared with wild foxes (the natural hosts) to vulnerable human populations (12). The same authors also conducted a survey in Chile, finding a higher number of dog–fox interactions at a coastal peri-urban site (60 interactions) compared to two other, less disturbed sites (12 interactions) (12). Societal culture, animal husbandry practices, dietary habits, education levels, conditions of poverty and social marginalization, the availability of potable water, and the rural or urban nature of the habitat are all variables that influence the epidemiological dynamics of various zoonoses (13). Populations in rural and peri-urban areas are in close contact with animals (often involving intensive rearing practices), resulting in significantly greater exposure to zoonotic diseases. Furthermore, the Pampas gray fox frequently ventures into these areas because of their proximity to its natural habitat. Consequently, the level of risk differs between urban and rural zones, although the recent expansion of gated communities has narrowed this gap. It is also worth noting that many canids in rural areas often feed on animal offal; this practice can increase the risk of pathogen transmission to other species—such as the Pampas gray fox—and even directly to humans. Similarly, in urban and peri-urban areas, population overcrowding increases the likelihood of disease transmission and epidemic outbreaks (13). In the context of territorial expansion, the creation of new neighborhoods in peri-urban areas has facilitated the transmission of various zoonotic diseases to the human population (13).

The presence of protozoa (*T. gondii* and *N. caninum*), bacteria (*Leptospira* spp., *Brucella* spp., and antimicrobial-resistant Enterococcus spp.), and viruses (rabies) illustrates how the Pampas gray fox facilitates pathogen transmission (10). Therefore, the Pampas gray fox should be viewed less as a simple reservoir and more as an indicator of ecological dysfunction, given that health risks are typically associated with transmission through uncontrolled domestic animals rather than the species itself (10). Therefore, the aim is to improve understanding of the spatiotemporal patterns of interspecific contact between invasive and native carnivores to help prevent the spread of various pathogens (12). Consequently, education is a fundamental component of preventing such transmission. It is worth noting that this education must be contextualized within the specific settings where pathogen transmission is likely to occur. Hence, it is important to raise awareness about zoonoses in the various spaces shared by humans and animals, as well as among different animal species. The interaction among animals, humans, and their habitats should be a central focus of modern scientific education; the goal is not only to teach the definitions and characteristics of various zoonoses but also to help people understand the environments in which transmission takes place. This enables the public to make informed decisions—for instance, on how to act when encountering animals.

In the case of the Pampas gray fox, human intervention is impacting its ecology through poisoning, hunting, road construction, and urbanization. This leads us to consider this animal as a model for understanding the capacity to govern shared life with integrity, maintaining ecological and health functions in balance (10). Therefore, education should encourage conservation by raising awareness of the human-caused threats affecting this animal and promoting potential solutions, such as banning the use of poisons, reducing wildlife road mortality, managing domestic animals, and fostering coexistence through a genuine “One Health, One Wellbeing” approach, as described by Vidal et al. (10), to achieve the integration of human, animal, and environmental health in urban planning.

The STS approach to One Health prevention education should incorporate the following elements, drawing on the book by Lampert (15):

- Mainstream the “One Health, One Wellbeing” or Conservation Medicine approach. In the case of the Pampas gray fox, education regarding the animal should not be conducted in isolation—treating the animal's physiology and habitat, zoonoses, and territorial/urban expansion as separate topics. Instead, the issue must be addressed through an integrated, cross-cutting approach that acknowledges its complexity.
- Contextualize instruction across different settings. Studying and understanding human–animal interactions differ depending on whether the setting is rural, peri-urban, or urban. Local wildlife should be identified, along with the ways in which the transmission of microorganisms between species may be altered.
- Teach about animal feeding practices. It is essential to understand the risks posed to the ecosystem by feeding domestic canids animal offal or raw meat that has not undergone proper sanitary inspection or food safety inspection.
- Develop educational initiatives grounded in health education, environmental education, and food education. In Argentina, it is important to note that environmental education and food education are mandated by law.
